# Study protocol; Thyroid hormone Replacement for Untreated older adults with Subclinical hypothyroidism - a randomised placebo controlled Trial (TRUST)

**DOI:** 10.1186/s12902-017-0156-8

**Published:** 2017-02-03

**Authors:** David J. Stott, Jacobijn Gussekloo, Patricia M. Kearney, Nicolas Rodondi, Rudi G. J. Westendorp, Simon Mooijaart, Sharon Kean, Terence J. Quinn, Naveed Sattar, Kirsty Hendry, Robert Du Puy, Wendy P. J. Den Elzen, Rosalinde K. E. Poortvliet, Jan W. A. Smit, J. Wouter Jukema, Olaf M. Dekkers, Manuel Blum, Tinh-Hai Collet, Vera McCarthy, Caroline Hurley, Stephen Byrne, John Browne, Torquil Watt, Douglas Bauer, Ian Ford

**Affiliations:** 1 Geriatric Medicine, Institute of Cardiovascular and Medical Sciences, University of Glasgow, Glasgow, UK; 2 Department of Public Health and Primary Care, Leiden University Medical Center, Leiden, Netherlands; 3 Department of Epidemiology and Public Health, University College Cork, Cork, Ireland; 4 Institute of Primary Health Care (BIHAM), University of Bern, Bern, Switzerland; 5 Department of General Internal Medicine, Inselspital, Bern University Hospital, Bern, Switzerland; 6 Leyden Academy on Vitality and Ageing, Leiden, Netherlands; 7 Center for Healthy Aging, University of Copenhagen, Copenhagen, Denmark; 8 Department of Internal Medicine, Leiden University Medical Center, Leiden, Netherlands; 9 Robertson Centre for Biostatistics, University of Glasgow, Glasgow, UK; 10 Institute of Cardiovascular and Medical Sciences, University of Glasgow, Glasgow, UK; 11 Department of Clinical Chemistry and Laboratory Medicine, Leiden University Medical Center, Leiden, The Netherlands; 12 Radboud University Medical Center, Nijmegen, The Netherlands; 13 Department of Cardiology, Leiden University Medical Center, Leiden, Netherlands; 14 Department of General Internal Medicine, Inselspital, Bern University Hospital, University of Bern, Bern, Switzerland; 15 Service of Endocrinology Diabetes and Metabolism, University Hospital of Laussanne, Lausanne, Switzerland; 16 School of Nursing and Midwifery, University College Cork, Cork, Ireland; 17 Department of Epidemiology and Public Health and Clinical Research Facility, University College Cork, Cork, Ireland; 18 School of Pharmacy, University College Cork, Cork, Ireland; 19 Department of Internal Medicine, Copenhagen University Hospital Herlev, Gentofte, Denmark; 20 Department of Medicine, University of California, San Francisco, USA; 21 Glasgow Royal Infirmary, Room 2.42, 2nd Floor New Lister Building, G31 2ER Glasgow, UK

**Keywords:** Subclinical hypothyroidism, Elderly, Randomised controlled trial, Levothyroxine, Quality of life, Thyroid disease

## Abstract

**Background:**

Subclinical hypothyroidism (SCH) is a common condition in elderly people, defined as elevated serum thyroid-stimulating hormone (TSH) with normal circulating free thyroxine (fT4). Evidence is lacking about the effect of thyroid hormone treatment. We describe the protocol of a large randomised controlled trial (RCT) of Levothyroxine treatment for SCH.

**Methods:**

Participants are community-dwelling subjects aged ≥65 years with SCH, diagnosed by elevated TSH levels (≥4.6 and ≤19.9 mU/L) on a minimum of two measures ≥ three months apart, with fT4 levels within laboratory reference range. The study is a randomised double-blind placebo-controlled parallel group trial, starting with levothyroxine 50 micrograms daily (25 micrograms in subjects <50Kg body weight or known coronary heart disease) with titration of dose in the active treatment group according to TSH level, and a mock titration in the placebo group. The primary outcomes are changes in two domains (hypothyroid symptoms and fatigue / vitality) on the thyroid-related quality of life questionnaire (ThyPRO) at one year. The study has 80% power (at *p* = 0.025, 2-tailed) to detect a change with levothyroxine treatment of 3.0% on the hypothyroid scale and 4.1% on the fatigue / vitality scale with a total target sample size of 750 patients.

Secondary outcomes include general health-related quality of life (EuroQol), fatal and non-fatal cardiovascular events, handgrip strength, executive cognitive function (Letter Digit Coding Test), basic and instrumental activities of daily living, haemoglobin, blood pressure, weight, body mass index and waist circumference. Patients are monitored for specific adverse events of interest including incident atrial fibrillation, heart failure and bone fracture.

**Discussion:**

This large multicentre RCT of levothyroxine treatment of subclinical hypothyroidism is powered to detect clinically relevant change in symptoms / quality of life and is likely to be highly influential in guiding treatment of this common condition.

**Trial registration:**

Clinicaltrials.gov NCT01660126; registered 8th June 2012.

**Electronic supplementary material:**

The online version of this article (doi:10.1186/s12902-017-0156-8) contains supplementary material, which is available to authorized users.

## Background

Subclinical hypothyroidism (SCH) is defined as an elevated serum thyroid-stimulating hormone (TSH) with normal serum levels of free thyroxine (fT4) and few or no hypothyroid symptoms [1]. Between 8 and 18% of adults over 65 years have biochemical features of SCH, the prevalence being higher among women [2]. SCH is a possible contributor to multiple problems in older age. From a biological point of view thyroid hormones have multiple pleiotropic effects, acting as an essential regulatory factor in numerous physiological systems, including the vascular tree and the heart [3], brain (including cognition and mood) [4], skeletal muscle and bone [5].

Subjects with SCH do not have the full symptom cluster of overt hypothyroidism; however they often report non-specific symptoms such as tiredness, dry skin, feeling cold, puffy eyes, constipation and cognitive problems [6]. Muscle symptoms such as cramps, weakness and myalgia are more common in SCH than in euthyroid controls [7]. Reduced exercise capacity in SCH may be due to impaired skeletal muscle function and increased oxygen requirements of exercise [8]. SCH may also cause a low-grade anaemia [9]. The most convincing epidemiological associations of SCH with disease states are with non-fatal and fatal coronary heart disease events [10].

In contrast to the epidemiological associations with poor health found in the whole population, SCH in very old subjects (≥85 years) might be associated with better health and survival compared to the euthyroid state, giving rise to the possibility of an age interaction for thyroid hormone effects [11].

The pituitary-thyroid axis is an evolutionary selected key regulator to optimize developmental processes and physiological responses to environmental stress. At the same time TH might have a major role in the settlement of the life-history trade-off between growth, metabolism, and inflammation axes, which is required for homeostasis maintenance. It is therefore feasible that survival beyond the reproductive phase is better off with lower levels of thyroid hormone signalling, away from the optimal characteristics that are generally required in the reproductive part of life, with a lower rate of aging as a result. Several experimental animal models are in agreement with this hypothesis [12].

Evidence is lacking about the benefits of levothyroxine replacement in the elderly with SCH, as no large RCT on the full range of relevant clinical outcomes have been performed [13]. The Cochrane systematic review of levothyroxine replacement for SCH summarised the evidence from RCTs up to 2006 [13]. It concluded that there was some evidence for improved cardiac function and blood lipids with levothyroxine replacement, but a lack of data for improved survival, reduced cardiovascular morbidity or improved health-related quality of life; data were available for only 350 patients in 12 RCTs, often of short duration (range 6–14 months).

Subsequent RCTs of levothyroxine as an intervention for patients with SCH (excluding during pregnancy) have not resolved the clinical uncertainty, with generally small and underpowered studies [4, 14–25]; the largest sample size was 120 patients [26], and the longest mean follow-up 14 months [27].

We are conducting a large RCT with power to detect clinically worthwhile benefits from levothyroxine replacement for SCH. Critical elements of the study design include recruitment of subjects with persisting SCH (excluding those in whom it is a temporary phenomenon, who are less likely to benefit), long-term follow-up, and a range of clinically important outcomes.

The primary outcomes are changes in 2 domains (hypothyroid symptoms and fatigue / vitality) on the thyroid related quality of life questionnaire (ThyPRO) [28] at 1 year (minimum follow-up). Secondary outcomes include general health-related quality of life (EuroQol) [29], fatal and non-fatal cardiovascular events, handgrip strength [30], executive cognitive function (Letter Digit Coding Test) [31], basic [32] and instrumental activities of daily living [33], haemoglobin, blood pressure, weight, body mass index and waist circumference. Patients are monitored for specific adverse events of interest including incident atrial fibrillation, heart failure and bone fracture. The study will also be used to establish a blood bio-bank, to be used in future research into causes and mechanisms of health, disease and disability in older people with SCH.

There are four main study objectives:

1. Does Levothyroxine treatment for SCH give multi-modal benefits for older people with SCH?
2. Are benefits seen across a wide range of outcomes, including prevention of cardiovascular disease, and improving health-related quality of life, muscle function and cognition?
3. Are benefits seen in specific subgroups of older people with SCH, including women, very elderly and those with mild degrees of SCH (TSH 4.6–10 mU/L)?
4. Are any benefits offset by adverse effects, such as atrial fibrillation or heart failure?

This manuscript represents the content of protocol version 6.1, 24^th^ March 2016.

## Methods

This is a randomised double-blind placebo-controlled parallel group trial of levothyroxine for older people with SCH, with a minimum 1 year of follow-up.

### Study population

The trial is running in four countries (UK, Ireland, the Netherlands and Switzerland). Potential subjects are identified from clinical laboratory databases and recruited from the community, aged ≥65 years with SCH, diagnosed on the basis of persistently elevated TSH levels (4.6–19.9 mU/L) with fT4 within the reference range. Baseline and follow-up TSH was measured by clinical laboratories affiliated with each clinical centre. TSH is measured on a minimum of two occasions at least three months apart, over a maximum period of 3 years. All subjects gave individual informed consent to participate (See Appendix 1 and 2; Additional files 1 and 2 for model patient information sheet and consent forms).

Exclusion criteria for the study:

i) Subjects currently prescribed Levothyroxine, anti-thyroid drugs, amiodarone or lithium.
ii) Recent thyroid surgery or radio-iodine (within 12 months).
iii) Grade IV NYHA heart failure.
iv) Clinical diagnosis of dementia.
v) Recent hospitalisation for major illness or elective surgery (within 4 weeks).
vi) Recent acute coronary syndrome, including myocardial infarction or unstable angina (within 4 weeks).
vii) Acute myocarditis or pancarditis.
viii) Untreated adrenal insufficiency or adrenal disorder.
ix) Terminal illness.
x) Patients with rare hereditary problems of galactose intolerance, the Lapp lactase deficiency or glucose-galactose malabsorption.
xi) Subjects participating in ongoing RCTs of therapeutic interventions (including Clinical Trials of Investigational Medicinal Products; CTIMPs).
xii) Plan to move out of the region in which the trial is being conducted within the next 2 years.

### Randomisation

Subjects are allocated to levothyroxine or placebo (1:1) with stratification by national site, sex and starting dose, using the method of randomly permuted blocks. The randomisation schedule is prepared by the data centre (Robertson Centre, Glasgow), independent of the clinical investigators. Implementation of the random allocation sequence is by an independent party, Mawdsley Brooks UK, who are responsible for packaging and labelling of the Investigational Medicinal Product (IMP) and masked to all other participant characteristics.

Enrolment of participants is done by study nurses, under the supervision of a medically trained principal investigator at each site. Allocation of study number is performed by central computer, with the decision triggered by the study nurses using a dedicated study website, following entering of eligibility data on an electronic clinic record form.

### Intervention

The IMP’s in this study are levothyroxine 25 and 50 micrograms tablets and identical placebo tablets for oral use. The tablets are white and round in shape with the tablet strength imprinted on the active and matched placebo tablets. All tables are identical in taste and smell. The tablets are manufactured and placed in blister strips by the supplier Merck, in accordance with Good Manufacturing Practice; distribution to national study sites is provided by Mawdsley Brooks UK.

The intervention starts with levothyroxine 50 micrograms daily (reduced to 25 micrograms in subjects <50Kg body weight or if known coronary heart disease – previous myocardial infarction or symptoms of angina pectoris) versus matching placebo for 6–8 weeks. Patients are advised to take their prescribed dose once daily in the morning before breakfast.

The dose is changed according to the serum TSH level as follows; 6–8 weeks after randomisation a blood sample is taken for serum TSH, with three possible actions:

1. TSH <0.4 mU/L: treatment dose reduced to 25 micrograms levothyroxine in those starting on 50 micrograms; reduced to 0 in those starting on 25 micrograms – blinding maintained by giving placebo matching the 25 micrograms dose; these patients will have a further check TSH after 6–8 weeks; if TSH remains <0.4 mU/L patient will be withdrawn from randomised treatment.
2. TSH ≥0.4 and <4.6 mU/L: no change to the treatment dose; patient to be reviewed at 12 months.
3. TSH remains elevated (≥4.6 mU/L): additional 25 micrograms levothyroxine, giving a total daily dose of 75 micrograms levothyroxine for those starting on 50 micrograms, or a total daily dose of 50 micrograms levothyroxine for those starting on 25 micrograms.

Titration of dose of levothyroxine (25 micrograms increments) against serum TSH is repeated up to a maximum of two occasions, at 6–8 week intervals, with a check of serum TSH performed at 6–8 weeks after all dose titrations. The investigators remained masked to the results of TSH measurements throughout the course of the study.

The above process (but with only a single up-titration) is repeated at 12 months (plus or minus 1 month) then annually (at 24 and 36 months plus or minus 1 month). The maximum possible dose of levothyroxine that is prescribed is 150 micrograms.

A mock titration (by computer algorithm) is performed in the placebo group aiming for approximately the same frequency as that likely to be required in the levothyroxine-treated group. We have adopted an adaptive schedule, in which the data centre allocates the same proportion of placebo patients to have dose adjustment (up and down) as are required in the levothyroxine group. This ensures that the burden of assessment, and number of tablets to be taken, is the same in both the levothyroxine and placebo groups. The computer generated allocation also ensures that the clinical investigators remain blind to treatment allocation.

Where a proposed up-titration of levothyroxine (or placebo) is thought to be clinically inappropriate (e.g. known non-adherence to IMP, recent major illness) the algorithm is manually ‘over-ridden’ and the patient is not up-titrated.

Criteria for withdrawal of participants on safety grounds:

- If overt biochemical hypothyroidism is identified (TSH ≥20 mU/L), the data centre requires a second TSH measurement with fT4 within two weeks; if overt biochemical hypothyroidism is confirmed (fT4 below reference range), the patient is required to stop the trial medication and recommended to attend GP for consideration of open treatment with levothyroxine.
- If biochemical hyperthyroidism (TSH < 0.4 mU/L) develops in the placebo group, or occurs at two consecutive follow-up visits in a patient in the levothyroxine group; i.e. persisting despite down-titration of the levothyroxine dose, the patient will stop the trial medication and attend their GP for consideration of further assessment and treatment of hyperthyroidism.

### Blinding

The study is double blinded. Subject blinding to treatment allocation is ensured through use of matched tablets for levothyroxine and placebo, with a mock titration in the placebo group (see above under intervention for details). Clinician and study centre blinding to treatment allocation is ensured by remote laboratory analysis of blood samples for TSH and dose titration as per computer algorithm.

All blood tests for in-study TSH and fT4 levels are performed by the research team without knowledge of the results; all results from the follow-up phase of the study are forwarded directly to the data centres who advise the clinical research team on any dose titration. The clinical research team are not informed of the results of thyroid function testing. The same process of blinding is followed for measurement of haemoglobin on a full blood count.

If the clinical investigator or attending physician deems that unblinding is necessary they will have 24-hr access to telephone unblinding through the data-centre. In the event of a SUSAR, the sponsor (but not the investigators) will be unblinded to facilitate reporting to the relevant regulatory authorities including the MHRA.

### Descriptive study data and outcome measures

Descriptive data to be recorded at screening and /or baseline are as follows:

1. Age, sex and race.
2. Lifestyle; smoking, alcohol intake.
3. Known cardiovascular disease, including history of coronary heart disease (angina pectoris or previous myocardial infarction), cerebrovascular disease (ischaemic stroke, transient ischaemic attack) or peripheral vascular disease (intermittent claudication), or any revascularisation procedure for ischaemic vascular disease; history of atrial fibrillation (AF); epilepsy; hypertension; diabetes mellitus; osteoporosis.
4. Prescribed medicines and over-the counter aspirin or non-steroidal anti-inflammatory drugs will be recorded at each study visit; medicine count will be used as an assessment of baseline co-morbidity.
5. Mini-mental state examination (MMSE) score [34] will be recorded at study baseline as a descriptor of general cognitive function. However it will not be repeated or used as an outcome measure as it is insensitive to change over the time-span planned for this study.
6. Home support services (e.g. home help, meals-on-wheels, district nursing) and home circumstances (e.g. living alone, co-habiting, standard or sheltered housing, or entry to care home), at study baseline and final review.

Primary study outcomes:

Initial plans were for joint primary study outcomes of thyroid-specific quality of life and of incident cardiovascular events. However, due to lower recruitment than initially planned, the study is underpowered to detect an effect on incident cardiovascular events and a formal modification to protocol (including on the registered trial protocol at clinicaltrials.gov) has been made to designate this as a secondary outcome.

Therefore the primary outcomes for the study are thyroid-related quality of life and symptom burden as measured by the ThyPRO hypothyroid symptoms score and fatigue / vitality score measured at study baseline, 6–8 weeks and 12 months post-recruitment and at final review [35]. The primary outcome is the change in the score between baseline and one year. For disease-specific and general quality of life, greatest effect will be expected after 1 year of treatment, and for these endpoints this will be the primary time-point for analysis. In a systematic review of thyroid related quality of life assessments ThyPRO was recommended as an appropriate assessment tool for benign thyroid diseases [35].

Secondary study outcomes:

- General health-related quality of life (EuroQol-5D, including the EuroQol visual anologue scale) [29]. Measured at study baseline, 6–8 weeks and 12 months post-recruitment and at final review.
- Comprehensive thyroid quality of life assessment ThyPRO-39 [28] - recorded at final follow-up.
- Fatal and non-fatal cardiovascular events (acute myocardial infarction; stroke; amputations for peripheral vascular disease; revascularisations for atherosclerotic vascular disease, including for acute coronary syndrome, and heart failure hospitalisations).
- Total mortality and cardiovascular mortality.
- Handgrip strength [30] (Jamar isometric dynamometer - best of 3 measures in dominant hand). Measured at study baseline, 12 months and at final review.
- Executive cognitive function (letter digit coding test; LDCT) [31]. Measured at study baseline, 12 months and at final review.
- Functional ability (basic Activities of Daily Living (ADL) measured using Barthel Index [BI] [36]; instrumental activities of daily living [33] measured using the older American resources and services [OARS] tool) [37]. Measured at baseline and final follow-up.
- Haemoglobin measured on a full blood count at baseline and 1 year.
- Blood pressure (systolic and diastolic). Measured at screening / baseline, 12 months and at final review. Measurements were made using an Omron 705IT, after a minimum of 3 min sitting, using a standard cuff (12–13 cm long and 35 cm wide) unless the arm is very small or large, when an appropriate alternative cuff size is determined by visual assessment.
- Weight, waist circumference and body mass index. Height, weight and waist circumference are measured at screening / baseline; weight and waist circumference are repeated at 12 months and at final review.

**Fig. 1:**
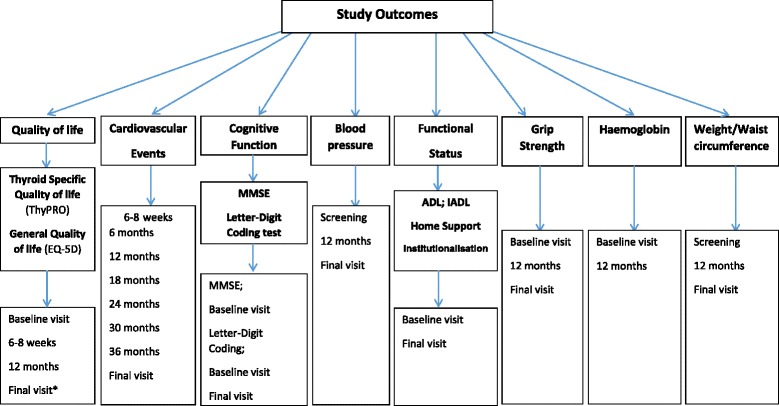
Outcome variables and timings of assessments

**Fig. 2:**
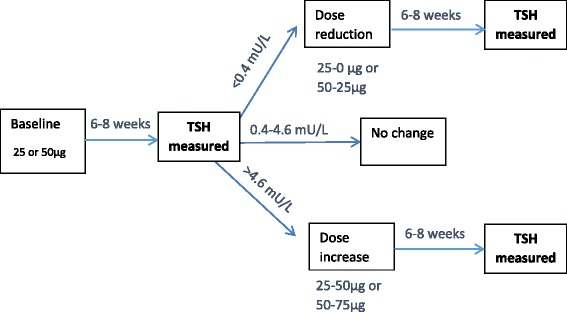
Levothyroxine dose titration flowchart. Levo-thyroxine dose is increased/decreased by 25 μg with a maximum dose of 150 μg. TSH checked 6–8 weeks after every dose change until TSH is within the reference range of 0.4–4.6 mU/L. If TSH is found to be ≥20 mU/L, a second TSH (and fT4) measurement is taken within 2 weeks. If a TSH of ≥20 mU/L is confirmed at second measurement then the patient is required to stop the trial medication and attend GP for further investigation

See Figs. 1 and 2 for summary of timings of each assessment.

Report forms for possible cardiovascular endpoints and SAEs are generated for the study nurses to complete; these are entered via the trial web portal which has an in-built notification to the Endpoints Committee. Adjudicated endpoints are entered via the trial web portal using separate adjudication record forms. Anonymised source documents are uploaded by the study nurses via the trial web portal, to assist in the adjudication process, in accordance with the committee’s requirements.

Drug accountability data is gathered for each patient including distribution date, quantity of study drug supplied, and drug supply returns including date, and quantity of tablets returned.

### Biobank

Blood samples for the study biobank are collected at baseline (40 ml venous blood) and at the 1 year visit (10mls venous blood). Baseline bloods are stored in 0.75 ml aliquots as follows - 3 EDTA plasma, 1 whole blood, 8 serum, 2 citrated plasma, 1 NaF plasma, 1 buffy coat, 3 heparin plasma; total: 19 aliquots per patient at baseline. One year bloods are stored in four 0.75 ml serum aliquots per patient.

The TRUST biobank is the communal property of the TRUST Executive Committee. The TRUST biobank is organised through the TRUST Executive Committee by the TRUST Biobank Committee. The TRUST Biobank Committee has a representative from each TRUST site and is approved by the TRUST Executive Committee. The partners in this collaboration are the partners associated with the trial’s principal investigators in Glasgow (Scotland), Cork (Ireland), Bern (Switzerland), and Leiden (Netherlands). All TRUST partners agree to the principles outlined below in order to permit the optimum information to be gained from exploitation of the Biobank.

The central location of the TRUST biobank is at the Department of Clinical Chemistry of Leiden University Medical Centre (LUMC), the Netherlands. The TRUST biobank consists of (1) all plasma, serum and DNA samples from randomised TRUST participants held either in the main biobank depository at LUMC or in the local laboratories where TRUST samples are handled during the study and (2) all data derived from analyses of these samples.

The Department of Clinical Chemistry of the LUMC is fully accredited by the Dutch Co-ordinating Committee for Quality Control of Laboratories in Health Care. Through the LUMC Department of Clinical Chemistry, the TRUST biobank adheres to all quality assurance standards that are necessary for a biobank. Furthermore, for all legal issues the TRUST biobank adheres to local (LUMC) and national guidelines. These guidelines cover donor rights, re-identification of donors, and process for removing samples from the biobank if a donor revokes consent.

Access to the material of the biobank is gained by submitting a proposal in writing to the chair of the Biobank Committee (PK). The chair will communicate the proposal to the other members of the Biobank Committee and together they will send a written proposal to the TRUST Executive Committee. Once the proposal is approved by the TRUST Executive Committee, the operational aspects of obtaining material will be handled by the Biobank group.

### Study recruitment and sample size justification

In the initial study plans we aimed to recruit 3000 community dwelling subjects aged 65 years or over with SCH. However due to a combination of factors we were unable to achieve this number; these included substantial delays in starting the study due mainly to difficulties in procuring suitable supplies of levothyroxine and matching placebo. Study recruitment targets were revised (October 2014; by when 290 patients randomised) with an anticipated minimum of 540 to be randomised; with an upscaling of geographical areas for recruitment in all countries we anticipated an increase up to a maximum of 750 patients randomised. Given the projections for recruitment, revised power calculations were calculated for minimum total recruitment number of 540 and a maximum number of 750.

Hypothyroid symptom and fatigue / vitality domains from the Thyroid-related Quality of Life patient-reported outcome measure (ThyPRO) [38] are our efficacy outcomes, and are given equal weights as co-primary outcomes, with the required p-value for statistical significance split equally to each (0.05/2 = 0.025 to each test). We assumed SDs for data at 1 year values (adjusted for baseline) of 13.3 and 18.3 (on 100-point scales) for hypothyroid and fatigue / vitality scales respectively. These SDs were justified on the basis of an interim analysis of data on the 8^th^ of January 2015. We calculated 80% power to detect a change with Levothyroxine treatment (versus placebo) of 3.5 or 3.0 points on the hypothyroid scale and 4.9 or 4.1 points on the fatigue / vitality scale with total sample sizes of 540 or 750 respectively. Personal communication from the author of ThyPRO (Torquil Watt) indicated that a 9 point change (100 point scale) would be a realistic and clinically meaningful effect size.

Retention of recruits was facilitated by offering flexibility in location of follow-up, and reasonable time windows (plus or minus 1 month for the 12 month and subsequent reviews) for review. Where patients elected to withdraw from active treatment they were offered follow-up, with recording of all study outcomes.

### Data and statistical analysis

The data centre for the study is based in the Robertson Centre for Biostatistics at the University of Glasgow. This sits in the Glasgow Clinical Trials Unit, a UK Clinical Research Collaboration fully registered Clinical Trials Unit. The Centre has an ISO 9001/2008 quality management system and TickIT, the corresponding standard for software development.

The statistical analysis plan is presented as an Additional file 3. Data will be summarised overall and by treatment group. Continuous variables will be summarised as number of observed values, number of missing values, mean and standard deviation, median and interquartile range and minimum and maximum. Categorical data will be summarised as number of observed values, number of missing values, number and percentage in each category.

Continuous efficacy outcomes involving measurement at follow-up and baseline will be analysed at each time point comparing treatment groups and adjusting for stratification variables and baseline levels of the same variable using linear regression. In addition, data items measured at more than one follow-up time will be analysed using repeated measures regression analyses and in terms of the final assessment for each participant. Continuous efficacy outcomes measured at final follow-up only will be compared between treatment groups using linear regression adjusting for stratification variables (site, sex and starting dose of levothyroxine).

For all efficacy outcomes measured at baseline and final visit, additional exploratory analyses that adjust for time from baseline to final visit will be carried out. Distributions of the residuals will be reviewed and will be taken into consideration in assessing whether or not additional analyses based on transformations should be carried out. When calculating ThyPRO scores, valid raw total scores containing missing items will be scaled so that the maximum possible score is maintained.

Time-to-event outcomes will be compared between groups using Cox proportional hazards regression models adjusting for stratification variables. Time to event curves will be based on the Kaplan-Meier method.

All efficacy analyses will be carried out on the intention to treat (ITT) population. Safety analyses will be carried out on the ITT population. The main analyses will be modified ITT based on participants with data on the outcome of interest. These analyses will be supported with sensitivity analyses using mixed effects models and multiple imputations. Primary and secondary analyses will be repeated on the per protocol population as exploratory analyses.

The primary and secondary outcomes at 12 months will also be analysed in the following subgroups by adding the subgroup variable and its interaction with treatment group:

- Sex (Male/Female)
- Baseline TSH
  - ○ <10 / ≥10
  - ○ <7 / 7–9.99 / ≥10

In addition, continuous interactions with age at randomisation and baseline TSH will be analysed analogously.

Study outcome analyses will be repeated on the per protocol population as exploratory analyses.

### Patient safety and adverse event recording

We have taken particular care in devising a titration algorithm to avoid any possibility of prolonged periods of thyroid hormone over replacement in those allocated to levothyroxine. This should substantially reduce the risks of adverse effects such as atrial fibrillation or cardiac failure. In epidemiological studies these problems are observed in association with biochemical hyperthyroidism and not with TSH within the reference range. Similarly for those allocated to placebo we have developed an algorithm that is designed to detect those who develop overt hypothyroidism are require to start open-label levothyroxine. These measures are designed to ensure safety of those who are randomised into the trial.

Within ThyPRO we are assessing the domain of hyperthyroid symptoms as a possible adverse effect (recorded at baseline, 6–8 weeks, 12 months and final visit).

Adverse events (AEs) are recorded, notified, assessed, reported, analysed and managed in accordance with the Medicines for Human Use (Clinical Trials) Regulations 2004 (as amended). Certain potential adverse events are anticipated or likely as a result of the study and study population. The adverse events detailed below are likely to occur in the context of over replacement of levothyroxine. Our dose titration scheme and study processes of careful monitoring of thyroid function tests are designed to ensure we avoid prolonged periods of thyroid hormone excess.

Full details of all AEs of special interest (new atrial fibrillation, heart failure, fractures, new diagnosis of osteoporosis) including the nature of the event, relationship to study drug and outcome are recorded in the electronic case report form. AEs of special interest are monitored and followed up until satisfactory resolution or stabilization.

#### Atrial fibrillation (AF)

AF is associated with subclinical hyperthyroidism [39] and therefore is a potential risk of thyroid over-replacement for SCH. It should not occur if TSH is maintained in the normal range, however we pay particular attention to identifying this possible adverse event.

Cardiac rhythm is determined at study baseline, and new onset AF, paroxysmal or persisting, is diagnosed from an annual single-lead electrocardiograph, or if noted on 12-lead electrocardiograph or telemetry performed as part of hospitalisation or other clinical care, or if identified by inquiry about hospitalisations and out-patient visits (at all patient contacts). This general process of screening for atrial fibrillation has been found to be very sensitive for identifying new cases [40].

We use a single-lead recorder (Omron HeartScan HCG-801-E). This provides a simple and quick assessment of cardiac rhythm; it has been shown to have high diagnostic accuracy for AF (sensitivity 99%, specificity 96%) compared to a standard 12-lead electrocardiograph [41].

#### Heart failure

Prevalent heart failure and incident heart failure diagnosis / hospitalisations will be recorded, as this outcome is a potential risk of thyroid dysfunction and of thyroid hormone over replacement [42].

#### Fracture

Musculoskeletal effects of Levothyroxine are described, including osteopenia/ osteoporosis [6]. We record all new fractures and all new diagnoses of osteoporosis. Formal screening for osteoporosis is not required for this trial.

### Study monitoring and auditing

Study monitoring visits are conducted according to a study-specific monitoring plan devised by NHS Greater Glasgow and Clyde and subsequent monitoring reports are reviewed by NHS Greater Glasgow and Clyde. At a minimum, each site was monitored before the study commenced (Study Initiation), study visit(s) and at the end of the trial (Study Close Out Visit). Additional monitoring visits were undertaken if required and the study was subject to routine or for-cause audit visits. Investigators and site staff were notified in advance of any audit and/or monitoring visits. Sponsors out-with the UK identified the level of monitoring required and developed a monitoring plan, informed by the UK monitoring plan.

### Committees, role of sponsor / funder

The committee structure for the study included the following. The main decision making group for the study is an Executive group of principle investigators (PIs) (Chair Professor David J Stott; members Professor Ian Ford, Professor Jacobijn Gussekloo, Professor Patricia M Kearney, Dr Nicolas Rodondi, Professor Rudi GJ Westendorp). The strategic planning and conduct of the study is supervised by a Steering Committee (Chair Professor David J Stott) which included the above PIs, plus key ancillary partners; the PIs approved additional members of the steering committee as required through the study. This committee provided a discussion forum for information to be shared, and opportunity given to influence the conduct of the study. National organising committees (chaired by the national PIs) supervised the conduct of the trial in each of the 4 participating countries.

The trial is subject to supervision by an Independent Data Monitoring Committee (IDMC). Members are Professor Gary Ford (Chair; Chief Executive Officer of the Oxford Academic Health Science Network, Oxford), Professor Thompson G Robinson (University Hospitals of Leicester NHS Trust, Department of Cardiovascular Sciences, Leicester Royal Infirmary, Leicester), Professor Colin Dayan (Institute of Molecular and Experimental Medicine, Cardiff University School of Medicine, Heath Park, Cardiff), Professor Kathleen Bennett (Department of Pharmacology and Therapeutics, Trinity Centre for Health Sciences, St James’s Hospital, Dublin). The primary responsibility of the IDMC is to assess safety data in order to protect the ethical and safety interests of the subjects recruited into the study, while safeguarding, as far as possible, the scientific validity of the study. The IDMC reviews information on the progress of the trial and selected safety and efficacy data accruing from the trial, but may request additional data if considered necessary in order to support the assessment of relative risk and benefit within the study.

Members of the study Endpoint Committee are Professor Peter Langhorne (Chair; Professor of Stroke Care, Institute of Cardiovascular and Medical Sciences, University of Glasgow), Professor J Wouter Jukema (Vice-chair; Professor of Cardiology, Leiden University Medical Center, The Netherlands), Dr Tin Hai Collett (Department of Ambulatory Care and Community Medicine, University of Lausanne, Switzerland), Prof. Olaf M Dekkers Leiden University Medical Center, The Netherlands) and Dr Anne Marie O’Flynn (Department of Epidemiology and Public Health, UCC, Ireland). The role of the Endpoint Committee is:

- To provide independent and unbiased review of clinical endpoint events which occur during the study.
- To ensure unified and unambiguous events evaluation practices across the study, through application of standardised event criteria, per protocol specifications.
- To compensate for regional diversity in medical practice in the area of endpoint evaluation and classification, thereby reducing the impact of this diversity.

This includes review and classification of all: causes of death, strokes, myocardial infarctions, heart failure hospitalisations, which are identified as potential clinical endpoints.

The classification of endpoints is based on data from a case report form and appropriate source documents. The EPC is blinded to treatment allocation.

There is a TRUST Biobank committee consisting of Professor Patricia M Kearney (chair), NS, H Anette van Dorland (Berne, Switzerland), WPJdenE. This committee advises the steering committee on storage and usage of the TRUST biobank.

The primary co-sponsors of the study are NHS Greater Glasgow and Clyde and University of Glasgow.

Prior to study initiation, a non-commercially funded clinical trial co-sponsorship agreement was put in place between NHS Greater Glasgow and Clyde and University of Glasgow. The role and liabilities each organisation will take under Clinical Trials Regulations, 2004 (SI 2004:1031) are laid out in this agreement signed by both organisations. The University of Glasgow is responsible for carrying out the obligations and responsibilities set out in the aforementioned agreement, and shall be deemed “sponsor” for the purposes of Part 3 of the regulations in relation to the study. NHS Greater Glasgow and Clyde shall be responsible for carrying out the obligations and responsibilities set out in the agreement, and shall be deemed “sponsor” for the purposes of Parts 4, 5, 6 and 7 of the regulations in relation to the study.

Each participating member/associated state has designated a legal entity to operate as national sponsor. The UK co-sponsors delegate sponsor responsibilities to each identified legal entity and this is defined in a ‘Research Agreement for the performance of an intergroup clinical trial’. A fully executed agreement was implemented with each participating member/associated state prior to the TRUST study starting in that country. The sponsors were responsible for ensuring appropriate indemnity and insurance is in place to cover the liabilities arising from trial conduct and participation. The sponsors of the study take no role in the preparation or approval of scientific outputs from this trial.

Merck KGaA have supported the study by providing (free of any cost or charges) the medication, in form of levothyroxine 50 microgram and 25 microgram tablets and matching placebos. All supplies are manufactured in accordance with current Good Manufacturing Practice and in accordance with EU Directive 2001/20/EC. Merck KGaA reserve the right to ask for deletion of any Merck confidential information. The views and opinions described in this paper do not necessarily reflect those of Merck KGaA.

### Dissemination

We are in a strong position to ensure effective dissemination of the results of the TRUST study, including early adoption into clinical practice. The patient advocacy group Thyroid Federation International will play a key role in planning the dissemination strategy and approving outputs to ensure methods and content fit with the public need. Publications are approved through the Steering Committee which involves representatives from all partner organisations. It is anticipated that the main study results will be announced at the Endocrine Society Meeting in Orlando 1-4th April 2017.

We have a strategy for informing participant patients and their general health care practitioners. At the final visit we determine what information the patient wishes to receive about their treatment in the study. Communication to the patient and GP about individual treatment is made normally within 15 working days after completion of the close-out visit. Information provided for the patient’s health care practitioner will include treatment allocation, final dose (if on levothyroxine) and TSH level at final visit. Unblinding of patient and their health care practitioner requires either a member of the study team or an independent medical advisor to be unblinded in the course of release of information; these person(s) are not involved in any way in measurement or recording of study endpoints or subsequent adjudication of SAEs (including gathering information or adjudication), either as possible vascular events or as events that are being considered for expectedness or severity for the MHRA or equivalent regulatory authority. This minimises the risk of observer bias and ensure the integrity of study data on SAEs is preserved.

We will provide access to the overall results of the study and explain implications for clinical practice to participating patients and for their health care practitioners, with national stakeholder meetings in country for supporting clinicians and patients; we also will announce the results using a study website. We aim for these communications about the results of the study to be within 1 month of the primary publication.

## Discussion

The TRUST trial is the first large-scale multicentre RCT of treatment of older people with SCH, comparing levothyroxine with matching placebo. The study will contribute substantially to knowledge on treatment of SCH in older people, with good statistical power to detect a meaningful effect on symptoms and thyroid-related health-related quality of life. We anticipate the main study results will be published in the Spring of 2017.

## Appendix 1

### **Patient information sheet for screening**

**Title of Project: Thyroid hormone replacement for subclinical hypothyroidism – the TRUST study.**

You are invited to attend a screening visit for a research study. The aim of the research is to determine if treating subclinical hypothyroidism with thyroxine gives health benefits to older people.

**What is subclinical hypothyroidism?**

Subclinical hypothyroidism means that the thyroid gland, which is found in your neck, may not be producing the right amount of thyroid hormones that your body needs to do its job well. Thyroid hormones are important as they help regulate different parts of the body, including the circulation, heart, muscle and brain.

Subclinical means that there are no noticeable symptoms or symptoms are at a level for which treatment is not certain to be beneficial. Without a blood test, it is impossible to know that you have subclinical hypothyroidism.

**What is the purpose of this screening?**

Subclinical hypothyroidism is a common condition among older men and women in Europe. A person may have subclinical hypothyroidism, and experience no symptoms. Subclinical hypothyroidism is not usually a serious health condition. The purpose of screening is to identify people who are suitable to take part in The TRUST Study. This is a study of the effects of thyroxine replacement for older people with subclinical hypothyroidism.

**The TRUST Study**

Some small studies have shown that when older people with subclinical hypothyroidism take thyroxine supplements their blood vessels become healthier and their heart function improves. However, there may be unwanted side effects of thyroxine supplements too, these may include an irregular heart beat (arrhythmia) and bone thinning (osteoporosis) which can lead to an increased risk of bone fracture.

The purpose of this study is to find out what effects (good and bad) of thyroxine replacement have on older people with subclinical hypothyroidism.

**Why have I been invited to take part?**

We are inviting people over the age of 65 who have had a recent blood test at their doctors the results of which suggest that they may have subclinical hypothyroidism and therefore may be suitable to take part in the TRUST Study. We have asked your GP or hospital doctor for permission to approach you about taking part in screening for the TRUST Study and they have agreed. However, it important to note that your GP or hospital doctor has no other involvement with this study.

**Do I have to take part?**

You are not in any way obliged to take part - it is up to you to decide. If you do decide to take part you will be asked to sign a consent form for screening for the study. Even if you decide to take part you are still free to withdraw at any time and without giving a reason. This will not affect the standard of care you receive.

**What will happen to me if I take part?**

If you decide that you are interested in taking part in screening, you will be asked to sign a consent form. Then you would be invited to a screening visit at your GP surgery or local hospital Clinical Research Facilities, at a time that suits you. However, you may prefer to be assessed at home and this can be arranged with the study nurse. The screening visit would take place up to six weeks before the study starts and will last up to 30 min. The purpose of screening is to decide if you are eligible to take part in the TRUST study. To decide this, you would be asked some medical questions, your blood pressure, height, weight and waist circumference would be measured and a blood test would be taken. We would also collect some information from your medical record (paper-based and computerised), held by your GP or hospital; examples include information about your diagnoses, lab results, medical procedures, and medications). This is because we need to know if you have any problems with your health.

If the results from the screening tests and medical record examination are satisfactory you would be invited to take part in the TRUST study. The blood results from this visit will be available within 1 week. If these confirm that you are suitable to take part in the study you will be sent further details by post including a written invitation to take part in the treatment phase of the study. You will be informed in writing if you are not suitable for entry into the treatment phase of the study, with the reasons explained. If we find a medical condition of which you are unaware (e.g. high blood pressure), we will inform you, and write to your GP.

If you are suitable to take part in the full study, and decide to take part, the drug that will be tested is Levothyroxine, given as a capsule and taken by mouth. The purpose of the TRUST study is to determine whether there are benefits and drawbacks of giving Levothyroxine to older people with subclinical hypothyroidism.

We will follow up the long-term health outcomes of all people who are screened for entry into the TRUST trial, whether or not they enter the treatment phase of the study. We plan to do this by looking at routinely recorded health information held by the NHS and records maintained by the General Register Office in Scotland. Your paper and computerised health records will be used by the University of Glasgow to follow up your future long-term health status. This will include paper and computerised records held by the NHS and electronic records maintained by the General Register Office at the Scottish Government.

If you are suitable to enter the full study, and decide to take part, the drug that will be tested is levo-thyroxine, given as a tablet orally (by mouth). We are testing whether it gives benefits to older people with a mildly underactive thyroid.

**Reasons we might not ask you to take part in the study**

If you are on certain drugs that affect thyroid blood tests or how the thyroid works, such as lithium, amiodarone, carbimazole or levothyroxine then it would not be appropriate for you to enter the study.

If your repeat thyroid blood tests show that your thyroid gland is now working normally, you then do not need any treatment and it would not be appropriate for you to enter the trial. On the other hand if your blood tests show your thyroid gland is now very underactive, then you will be advised to discuss these results with your GP, and it is likely you will require thyroid hormone treatment; it would not then be appropriate for you to enter the study.

**Long-term follow-up of your health**

We will follow up the long-term health outcomes of all people who are screened for entry into the TRUST trial, whether or not they enter the treatment phase of the study. We plan to do this by looking at routinely recorded health information held by the NHS and records maintained by the General Register Office in Scotland. Your paper and computerised health records will be used by the University of Glasgow to follow up your future long-term health status. This will include paper and computerised records held by the NHS and electronic records maintained by the General Register Office at the Scottish Government.

**Will my expenses be paid?**

Travel expenses will be provided to cover the cost of attending your visit.

**Will my taking part in this study be kept confidential?**

All information which is collected about you will be kept strictly confidential. We will keep your personal details on a secure computer at Glasgow University and the Clinical Research Facility. This is required to allow us to communicate with you (e.g. by post or telephone), and for us to link to the routine health information that is held by the Scottish Government.

However your name and contact details will not be disclosed to any other people, other than to your GP or hospital doctor who is providing care to you. Your GP and any hospital doctors who are caring for you will be notified of your screening for participation in the trial. Agreement from you that your GP can be informed is a condition of entering the study.

Any information which leaves your GP surgery or hospital will have your contact details, including name and address, removed so that you cannot be recognised from it. Your anonymised details and results will be shared with our collaborating investigators in the Netherlands, Ireland, Switzerland and the USA.

**Who is organising and funding the research?**

The organisation funding the research is the European Union. The team conducting the research in Scotland is from the University of Glasgow (linking with Greater Glasgow Health Board); this research is in collaboration with Leiden University Medical Centre in the Netherlands, Cork in Ireland, and University of Berne in Switzerland. We are also collaborating with thyroid experts from the University of California in the USA.

**Contacts for Further Information**

Further information can be obtained through the study freephone 0800 328 0772, or on the study website (www.trusthyroidtrial.com).

General information about thyroid problems can be found on the Thyroid Federation International website at www.thyroid-fed.org.

The doctors who are running the clinical aspects of the study in Scotland are;

Professor David J Stott and Dr Terence J Quinn,

Academic Section of Geriatric Medicine,

Institute of Cardiovascular and Medical Sciences, University of Glasgow,

4^th^ floor Walton building Glasgow Royal Infirmary G4 0SF.

## Appendix 2

### **Participant information sheet for randomisation**

**Patient information sheet for the trust study**

**Thyroid hormone replacement for subclinical hypothyroidism – the TRUST study.**

**Invitation paragraph:**

You are being invited to take part in a research study. Before you decide whether or not to take part it is important for you to understand why the research is being done and what it will involve. Please take time to read the following information carefully and discuss it with friends, relatives and your GP if you wish. Ask us if there is anything that is not clear or if you would like more information. Thank you for reading this information sheet.

**Background**

You have been asked to take part in this study because your recent screening blood test has told us that you have subclinical hypothyroidism. Subclinical hypothyroidism means that the thyroid gland, which is found in your neck, is not producing the right amount of thyroid hormones that your body needs to do its job well. Thyroid hormones are important as they help regulate different parts of the body, including the circulation, heart, muscle and brain. Subclinical means that there are no noticeable symptoms. Without a blood test, it is impossible to know that you have subclinical hypothyroidism. Subclinical hypothyroidism is a common condition among older men and women affecting up to 1 in 6 of over-65s.

**How many people like me (participants) will be taking part in this study?**

There would be up to 750 people enrolled in this study from Scotland, Ireland, The Netherlands and Switzerland.

**What is the purpose of the study?**

We aim to determine whether treating subclinical hypothyroidism with Levothyroxine gives health benefits to older people. Small studies have reported less fatty lining in blood vessels (atherosclerosis) and improved heart function with medication (Levothyroxine), but no large clinical trials have been performed. However, there may be unwanted side effects of Levothyroxine supplements too, these include an irregular heart beat (arrhythmia) and bone thinning (osteoporosis) which can lead to an increased risk of bone fracture.

The purpose of this study is to find out what effects (good and bad) of Levothyroxine replacement have on older people with subclinical hypothyroidism. We are interested in how well Levothyroxine works to:

- prevent heart and circulatory system problems (such as stroke)
- improve muscle function (such as strength)
- improve health related quality of life (by reducing symptoms such as tiredness)
- improve speed of thinking

You would be monitored carefully to ensure that you are not over-medicated with Levothyroxine; we believe this is important to reduce the risk of any side effects.

**Why have I been chosen?**

You have been chosen to be invited to take part in this study as your blood tests have shown that you have a mildly underactive thyroid gland with no symptoms or with symptoms at a level that treatment is not certain to be beneficial, and you are 65 years of age or older. You have had a screening assessment that has shown that your thyroid blood tests remain mildly underactive and you are suitable to enter the study.

**Do I have to take part?**

You are not in any way obliged to take part - it is up to you to decide. If you do decide to take part you will be asked to sign a consent form. Even if you decide to take part you are still free to withdraw at any time and without giving a reason. This will not affect the standard of care you receive.

**If I agree to take part, will I get the Levothyroxine drug?**

Once you have completed all the baseline information you will be assigned either to receive daily Levothyroxine, or to receive a placebo. A placebo is a pill which looks, tastes and smells like the real thing but is not; it contains no active ingredient. You will be assigned to one group or the other using a process of randomization. Randomization means that you have the same chance of being assigned to the Levothyroxine group or the placebo group. It is like tossing a dice or flipping a coin. This helps to make sure that the patients in each group (Levothyroxine or placebo) of the study are roughly the same, so that we can be sure that we get the correct answer to the question that the study is asking. If you chose to enter the study you would have a 50:50 chance of getting Levothyroxine or placebo. A computer will choose which group you are put in. Neither you, the doctors, study nurses or any of the people involved in the study will choose or know what group you will be in (although, if your doctor needs to find out he / she can do so).

**What else will happen to me if I take part?**

The first (baseline) study visit will be at your GP surgery or at your local hospital Clinical Research Facilities (if it suits you better, this can be done at your own home). We anticipate this visit will last for 60–90 min.

This assessment will include:

- Check of any new medical problems, changes to your medicines or home circumstances.
- A heart rhythm check, using an electronic recorder. To do this the research nurse will instruct you to hold a recording box in one hand, and press it gently over the front-left side of your chest for 30 s.
- Assessment of your quality of life, using 2 thyroid symptom questionnaires and a general quality of life questionnaire.
- Measurement of your grip strength; you will be asked to squeeze a portable grip measure machine as hard as you can, 3 times in succession.
- A 30-point memory questionnaire.
- Measurement of your speed of thinking, using a paper exercise taking 1 min.
- Assessment of how you are managing in everyday activities, using an Activities of Daily Living questionnaire, and how you are managing socially using a short questionnaire.

A blood sample (equivalent to 8 teaspoons) will then be taken to measure your blood count and to store blood for future laboratory analysis of factors that predict future illnesses; this will include storage of your genes (DNA) to allow us to examine any inherited factors that lead to illness in later life. A further small sample (equivalent to 3 teaspoons) will be repeated one year later, and stored for future research on the effects of thyroid hormone. You have the option of opting out of this part of the study if you wish.

Some of the above measures (general quality of life, speed of thinking and grip strength) and taking extra blood for future laboratory analysis will only be done in some of the research sites – so you may not be offered these tests.

You will be reviewed face-to-face by the study nurses at 6 to 8 weeks and 12 months then annually up to a maximum of 3½ years. In addition the study nurses will contact you by telephone at interim periods; 6, 18 and 30 months. It is anticipated that the review visits will take around 30 min, and telephone calls 15 min.

At all of these contacts the study nurses will enquire about any new illnesses or changes to your usual medicines. At 6 to 8 weeks, 12 months, and annually thereafter, a blood test (volume 2 teaspoons) will be taken to check your thyroid levels. This is to ensure that if you are treated with levothyroxine we give you the right dose; if you receive placebo we will know that your thyroid is not slowing down further – if this occurs you will require review by your GP. If your thyroid tests need adjustments to be made to your dose of tablets you will be asked to return for a further blood test in 6 to 8 weeks. A member of the research team may contact you via telephone to confirm that you have received the study medicines.

- The heart rhythm check will be repeated annually and at your final visit.
- The quality of life measurements will be repeated at 6 to 8 weeks and at 12 months and at your final visit.
- The hand-grip measure and blood pressure (blood pressure was checked at screening visit) will be repeated at 12 months and at your final visit.
- Your weight and waist circumference which was checked at the screening visit will be repeated at 12 months and at your final visit
- The speed of thinking paper exercise, and questionnaires on activities of daily living, social function, and home circumstances and support will be repeated at your final visit.

Travel expenses will be provided to cover the costs of attending all your visits.

The total duration of the study is 5 years (finishing in 2017). If you take part in the study your personal participation will be for at least 1 year, and could be as long as 3 ½ years (42 months).

**What do I have to do?**

During the study you will be asked to take between 1 and 3 tablets, once every day, and attend the study visits as listed above, including reporting any illnesses or side effects. There are no lifestyle restrictions or dietary restrictions from taking part in this study. You will still be able to drive, take part in sport, or give blood, should you wish to do so. During the study you should continue to take your regular medication.

**What is the drug that is being tested?**

The drug that is being tested is Levothyroxine, given as a tablet orally (by mouth). This is a hormone that occurs naturally in the body. It is the standard treatment when there is no doubt that the thyroid gland is underactive. Levothyroxine is used by between 3 and 5% of the population. We are testing whether it gives benefits to older people with a mildly underactive thyroid.

We will start the treatment with a low dose either 25ug or 50ug. The dose will be reviewed at 6 to 8 weeks and 12 months then annually. Adjustments will be made depending on a thyroid blood test; some people will require an increase, and some a decrease. Over the course of the study the dose will be increased to a maximum of 3 tablets per day (150ug). Similar adjustments will be also be made for some people in the placebo group, to ensure that people do not guess which group they have been allocated to.

**What are the alternatives for treatment?**

The main options for people with a mildly underactive thyroid gland are either to start Levothyroxine tablets, or to be followed up with no treatment. In either case a regular (e.g. annual) blood test is usually required.

**What are the possible side effects of Levothyroxine?**

Side effects from levothyroxine treatment are only rarely seen, particularly if blood tests are done regularly and adjustments to Levothyroxine dose are made to keep thyroid hormone levels in the normal range. If Levothyroxine is prescribed in too high a dose there is a risk of side effects. These could include development of an irregular heart rhythm called atrial fibrillation. This can cause tiredness, shortness of breath and an increased risk of stroke. Increased strain on the heart with excessive thyroxine doses could worsen angina (chest pain coming from the heart) or cause breathlessness and leg swelling. If you are overmedicated with thyroxine there may be an increased risk of thinning of bones (osteoporosis) and fractures.

If you are on an anticoagulant it is possible that the dose may need to be adjusted to prevent your blood becoming too thin. If you have diabetes there is a possibility that starting on levothyroxine might require an increase in dosage requirements of insulin or other anti-diabetic therapy. If you are taking anti-depressant tablets there is a possibility of abnormal heart ryhthms.

However these problems are unlikely so long as we do regular blood tests to ensure that we do not overmedicate you.

For those allocated to the inactive tablets (placebo) there is a risk that their thyroid gland may slow down further, and they may develop symptoms of an underactive thyroid, including tiredness and lethargy.

Taking blood samples may cause slight discomfort and there may be a small chance of local bruising afterwards.

**What are the possible disadvantages and risks of taking part?**

If you have private medical insurance you should check with the company before agreeing to take part in the trial. You will need to do this to ensure that your medical insurance will not be affected.

**What are the possible benefits of taking part?**

Benefits to individual patients who take part in this study cannot be guaranteed. Even if it does not help you directly, the information we get from this study should help us make better decisions on treatment of future patients with subclinical hypothyroidism. If during the study, we find a medical condition of which you are unaware (e.g. high blood pressure), we will inform you and we will write to and inform your GP.

**What if new information becomes available?**

Sometimes during the course of a research project, new information becomes available about the treatment that is being studied. If this happens, your research doctor will tell you about it and discuss with you whether you want to continue in the study. If you decide to withdraw your research doctor will make arrangements for appropriate care to continue through your GP. If you decide to continue in the study you will be asked to sign an updated consent form.

Also, on receiving new information your research doctor might consider it to be in your best interest to withdraw you from the study. If this happens he / she will explain the reasons and arrange for your care to continue through your GP.

**What happens when the research study stops?**

When the research study stops your supply of study medicines will finish, and you will return any unused capsules to the research nurse. It is likely that the full results will not be available for several months. You and your GP will then be informed in writing of the key results of the study, and also which group you were in (Levothyroxine or placebo capsules).

After the study finishes there will then be a decision to make as to whether you then take Levothyroxine tablets as part of your routine care. This will be your decision, which you should make in consultation with your GP.

The study freephone will continue to be available for a minimum of 6 months after the study finishes; you will be able to use this to arrange to speak to one of the research doctors for further information or advice, should you wish.

After the study finishes the research team will continue to gather routine healthcare information about your hospital admissions and diagnoses, to enable further research to be done on long-term health outcomes in older people with subclinical hypothyroidism.

**What if something goes wrong?**

There are no special compensation arrangements if something goes wrong when you are taking part in this research project however you will receive the same legal protection as a standard National Health Service patient. If you are harmed due to someone’s negligence, then you may have grounds for legal action but you may have to pay for it. Regardless of this, if you wish to complain about any aspect of the way you have been approached or treated during the course of this study, the normal National Health Service complaints mechanisms will be available to you.

**Will my taking part in this study be kept confidential?**

All information which is collected about you will be kept strictly confidential. We will keep your personal details on a secure computer at Glasgow University and the Clinical Research Facility. This is required to allow us to communicate with you (e.g. by post or telephone), and for us to link to the routine health information that is held by the Scottish Government.

However your name and contact details will not be disclosed to any other people, other than to your GP or hospital doctor who is providing care to you. Your GP and any hospital doctors who are caring for you will be notified of your screening for participation in the trial. Agreement from you that your GP can be informed is a condition of entering the study.

Any information which leaves your GP surgery or hospital will have your contact details, including name and address, removed so that you cannot be recognised from it. Your anonymised details and results will be shared with our collaborating investigators in the Netherlands, Ireland, Switzerland and the USA.

**What will happen to the results of the research study?**

The results of the main part of the research should be available within 6 months of you completing the study, and it is expected the primary publication will be within 12 months. You and your GP will be informed in writing of the key results of the study (anticipated between 6 and 12 months after you finishing in the study), and also which group you were in (thyroxine or placebo tablets). The results and details of publications will also be placed on the study website, at the time of publication. You will not be identified in any report or publication.

**Who is organising and funding the research?**

The organisation funding the research is the European Union. The team conducting the research in Scotland is from the University of Glasgow (linking with Greater Glasgow Health Board); this research is in collaboration with Leiden University Medical Centre in the Netherlands, Cork in Ireland, and University of Berne in Switzerland. We are also collaborating with thyroid experts from the University of California in the USA.

**Who has reviewed the study?**

The research project has been reviewed by, and received approval from the following;

- The European Union grant reviewers (includes ethical review).
- The Scottish Multicentre Research Ethics Committee (MREC A).
- Thyroid Federation International, the umbrella organisation for patient support groups for those with thyroid disorders. It aims to work for the benefit of those affected by thyroid disorders throughout Europe and the world.

**Contacts for Further Information**

Further information can be obtained through the study freephone 0800 328 0772or on the study website (www.trusthyroidtrial.com). General information about thyroid problems can be found on the Thyroid Federation International website at www.thyroid-fed.org. The doctors who are running the clinical aspects of the study in Scotland are; Professor David J Stott and Dr Terence J Quinn, Academic Section of Geriatric Medicine, Institute of Cardiovascular and Medical Sciences, University of Glasgow, 4^th^ floor Walton building Glasgow Royal Infirmary G4 0SF.

## Additional files

Additional file 1: Participant consent form for screening. (DOC 79 kb)
Additional file 2: Patient consent form for randomisation. (DOC 64 kb)
Additional file 3: Statistical analysis plan. (DOCX 45 kb)

## Abbreviations

AE: Adverse event
AF: Atrial fibrillation
fT4: Free thyroxine
ITT: Intention to treat
LDCT: Letter digit coding test
MMSE: Mini-mental state examination
RCT: Randomisation controlled trial
SAE: Serious adverse event
SCH: Subclinical hypothyroidism
ThyPRO: Thyroid-related quality of life measure
TRUST: Thyroid hormone Replacement for Untreated older adults with Subclinical hypothyroidism - a randomised placebo controlled Trial
TSH: Serum thyroid-stimulating hormone
