# Supplementary material for: Study protocol; Thyroid hormone Replacement for Untreated older adults with Subclinical hypothyroidism - a randomised placebo controlled Trial (TRUST)
Source: BMC Endocr Disord. 2017 Feb 3;17:6. doi: 10.1186/s12902-017-0156-8 (PMC5291970; doi:10.1186/s12902-017-0156-8)
Supplement: Additional file 1: — Participant consent form for screening. (DOC 79 kb) [file 12902_2017_156_MOESM1_ESM.doc]

APPENDIX 2. Participant consent form for screening.


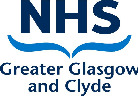

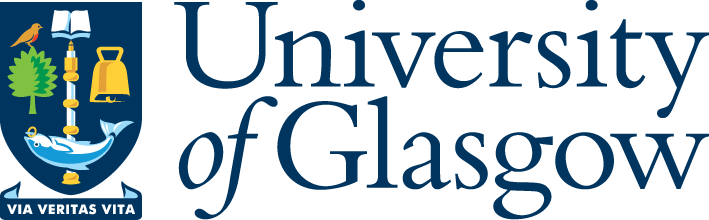


| Country: UK version 4.0 7^th^ August 2014  Screening Number:  **CONSENT FORM FOR SCREENING FOR RESEARCH STUDY**  **Title of Project: Thyroid hormone replacement for subclinical hypothyroidism – the TRUST study.**  Name of Researcher:   | **Please initial  to confirm** | | | | --- | --- | --- | |  | I confirm that I have read and understand the information sheet dated ......................... (version ...) for screening for the above study. |  | |  | I have had the opportunity to consider the information, ask questions and have had these answered satisfactorily. |  | |  | I understand that my participation is voluntary and that I am free to withdraw at any time, without giving any reason, without my medical care or legal rights being affected. |  | |  | I agree that relevant sections of any of my medical notes and data collected for screening for this study may be looked at by responsible individuals from the University of Glasgow, NHS Greater Glasgow & Clyde and from the regulatory authorities where it is relevant to my taking part in this research. I give permission for these individuals to have access to my records including from primary care, secondary care and any electronic records including prescribing and dispensing and laboratory data as described on the patient information sheet. |  | |  | I agree that paper and computerised records held by the NHS and electronic records maintained by the General Register Office may be used by the University of Glasgow to follow up my future health status linking up these different sets of information. |  | |  | I agree to my GP being informed of my screening for participation in the study. |  | |  | I agree to be screened for possible participation in the above research study. |  | | | |
| --- | --- | --- | --- | --- | --- | --- | --- | --- | --- | --- | --- | --- | --- | --- | --- | --- | --- | --- | --- | --- | --- | --- | --- | --- | --- | --- |
| _________________________ Name of Patient | ______________ Date | __________________________ Signature |
| __________________________ Name of person taking consent (if different from researcher) | ______________ Date | __________________________ Signature |
| __________________________ Researcher | ______________ Date | __________________________ Signature |
| When complete, 1 copy for patient: 1 copy for researcher site file: 1 (original) to be kept in medical notes. | | |
