# Supplementary material for: Study protocol; Thyroid hormone Replacement for Untreated older adults with Subclinical hypothyroidism - a randomised placebo controlled Trial (TRUST)
Source: BMC Endocr Disord. 2017 Feb 3;17:6. doi: 10.1186/s12902-017-0156-8 (PMC5291970; doi:10.1186/s12902-017-0156-8)
Supplement: Additional file 2: — Patient consent form for randomisation. (DOC 64 kb) [file 12902_2017_156_MOESM2_ESM.doc]

**Appendix 4. Patient consent form for randomisation.**

Country: UK version 4.0 August 2014

Patient Identification Number for this trial:

**CONSENT FORM FOR RESEARCH STUDY**

**Title of Project: Thyroid hormone replacement for subclinical hypothyroidism – the TRUST study.**

| Name of Researcher:  **Please initial   to confirm** |
| --- |
| I confirm that I have read and understood the information sheet dated. . . . . . . . . . . . . .  (version. . .) for the above study.  I have had the opportunity to consider the information, ask questions and have had these  answered satisfactorily.  I understand that my participation is voluntary and that I am free to withdraw at any time,  Without giving any reason, without my medical care or legal rights being affected.  I understand that relevant sections of my medical notes and data collected during the  Study may be looked at by responsible individuals from the University of Glasgow, NHS  Greater Glasgow and Clyde and the regulatory authorities, where it is relevant to my taking  Part in this research. I give permission to these individuals to have access to my records.  I agree to provide a blood sample for storage of my genes (DNA) for future research on  inherited factors contributing to ill health in later life.  I agree to provide a further small sample after one I year for storage for future research on  the effects of thyroid hormone.  I agree to my GP being informed of my participation in this study.  I agree to take part in the above research study.  Name of Patient Date Signature  Name of Person Taking consent  (if different from researcher) Date Signature  Researcher Date Signature  When complete, 1 copy for patient: 1 copy for researcher site file: 1 (original) to be kept in medical notes.   | ­­­­­­­­ |  |  | | --- | --- | --- | |  |  |  | | _______ Researcher |  |  | | When  notes. | | | |  | | | |  | | | |
