# Supplementary material for: Study protocol; Thyroid hormone Replacement for Untreated older adults with Subclinical hypothyroidism - a randomised placebo controlled Trial (TRUST)
Source: BMC Endocr Disord. 2017 Feb 3;17:6. doi: 10.1186/s12902-017-0156-8 (PMC5291970; doi:10.1186/s12902-017-0156-8)
Supplement: Additional file 3: — Statistical analysis plan. (DOCX 45 kb) [file 12902_2017_156_MOESM3_ESM.docx]

TRUST

Statistical Analysis Plan

Final Analysis

| Study Title: | Multi-modal effects of Thyroid hormone Replacement for Untreated older adults with Subclinical hypothyroidism; a randomised placebo-controlled Trial | | |
| --- | --- | --- | --- |
| Short Title: | TRUST | | |
| EudraCT Number: | 2011-004554-26 | | |
| Funded by: | European Commission  FP7-HEALTH-2011 (Seventh EU Framework Programme)/278148 | | |
| Protocol Version: | 6.0 | Date: | 19/01/2016 |
|  |  | | |
| SAP Version: | v1.0 | Date: | 22/04/2016 |
|  |  |  |  |

|  |  | Signature |  | Date |
| --- | --- | --- | --- | --- |
|  | |  |  |  |
| Prepared by: | Dr Martina Messow |  |  |  |
|  | Consultant Statistician  Robertson Centre for Biostatistics  University of Glasgow | | | |
|  |  |  |  |  |
| Approved by: | Prof Ian Ford |  |  |  |
|  | Director  Robertson Centre for Biostatistics  University of Glasgow | | | |
|  |  |  |  |  |
|  | Prof David Stott |  |  |  |
|  | Department of Academic Geriatric Medicine  University of Glasgow |  |  |  |
|  |  | | | |
|  |  |  |  |  |
|  |  |  |  |  |

**CONTENTS**

[1. Introduction 3](#_Toc445828980)

[1.1. Study Background 3](#_Toc445828981)

[1.2. Study Objectives 3](#_Toc445828982)

[1.3. Study Design 3](#_Toc445828983)

[1.4. Sample Size and Power 3](#_Toc445828984)

[1.4.1. Initial sample size calculation 3](#_Toc445828985)

[1.4.2. Revised sample size calculation 3](#_Toc445828986)

[1.5. Study Population 4](#_Toc445828987)

[1.5.1. Inclusion Criteria 4](#_Toc445828988)

[1.5.2. Exclusion Criteria 4](#_Toc445828989)

[1.6. Statistical Analysis Plan (SAP) 4](#_Toc445828990)

[1.6.1. SAP Objectives 4](#_Toc445828991)

[1.6.2. General Principles 5](#_Toc445828992)

[1.6.3. Current Protocol 6](#_Toc445828993)

[1.6.4. Software 6](#_Toc445828994)

[2. Analysis 6](#_Toc445828995)

[2.1. Study Populations 6](#_Toc445828996)

[2.2. Visit Attendance 7](#_Toc445828997)

[2.3. Baseline Characteristics 7](#_Toc445828998)

[2.4. Efficacy Outcomes 8](#_Toc445828999)

[2.4.1. Primary Outcome 8](#_Toc445829000)

[2.4.2. Secondary Outcomes 8](#_Toc445829001)

[2.5. Safety Outcomes 10](#_Toc445829002)

[2.5.1. Study Treatment 10](#_Toc445829003)

[2.5.2. Serious Adverse Events 11](#_Toc445829004)

[2.5.3. Other safety data 11](#_Toc445829005)

[3. Document History 12](#_Toc445829006)

[4. Tables and Figures 12](#_Toc445829007)

[5. Listings 12](#_Toc445829008)

1. Introduction
   1. Study Background

Subclinical hypothyroidism (SCH) is a common finding in older people across Europe, and is a likely contributor to multiple problems in older age. There is the potential for multisystem benefits from treatment of SCH with Levothyroxine.

- 1. Study Objectives

To test the efficacy of thyroxine replacement for subclinical hypothyroidism (SCH) in adults aged 65 years and older.

- 1. Study Design

Randomised double-blind placebo-controlled parallel group trial.

- 1. Sample Size and Power
     1. Initial sample size calculation

To detect a hazard ratio (HR) of 0.75 would need 379 events in total for 80% power at the 5% level (2-tailed). It is likely that drop-ins/drop outs will reduce the intention-to-treat effect. The drop-in and drop-out rates are each estimated to be less than 5% at 1 year and less than 10% at the end of the study. If the treatment effect is attenuated to a HR of 0.79 then 565 events in total would give 80% power. With an average follow-up of 3 years, observations from a similar age and risk population give an expected cardiovascular event rate of around 21%. 3000 subjects (1500 Levothyroxine, 1500 placebo) would have to be recruited.

- - 1. Revised sample size calculation

Given the projections for recruitment during the study, it became clear that it would not be possible to accrue the required number of events to achieve the above power. Event-free survival has therefore been dropped as a primary outcome and change in disease specific QOL (ThyPRO) hypothyroid function and tiredness are now co-primary outcomes.

Power calculations were revised for total recruitment numbers of 540 and 750, and with mean follow-up of 18 months. The global significance level is split equally between the two co-primary outcomes. A 9 unit change is a realistic and clinically meaningful effect size.

The addendum to the protocol v6.0 (page 60) states:

“Observed SDs for data at visit 5 (1-year) values adjusted for baseline are 13.3 and 18.3 (on 100-point (%) scales) for hypothyroid and tiredness scales respectively.

We will have 80% power to detect a change with levothyroxine treatment of 3.5% (3.0%) on the hypothyroid scale with total sample sizes of 540 (750).

We will have 80% power to detect a change of 4.9% (4.1%) on the tiredness scale with total sample sizes of 540 (750).”

- 1. Study Population

Potential subjects will be identified from clinical laboratory databases as having biochemical features consistent with SCH, (thyroid stimulating hormone [TSH] of >4.6 and <19.9 mU/L plus free thyroxine levels within the laboratory reference range).

- - 1. Inclusion Criteria

Community-dwelling subjects aged ≥65 years with SCH, diagnosed on the basis of elevated TSH levels (≥4.6 and ≤19.9 mU/L) and free thyroxine levels (fT4) within the laboratory reference range, measured on a minimum of two occasions at least 3 months apart (in Switzerland there was a minor variation the main study protocol - patients required only one measure of fT4 before recruitment).

- - 1. Exclusion Criteria
- Subjects currently on (anti)thyroid drugs, amiodarone or lithium;
- Recent thyroid surgery or radio-iodine;
- Grade IV NYHA heart failure;
- Prior clinical diagnosis of dementia;
- Recent hospitalisation for major illness;
- Recent acute coronary syndrome;
- Acute myocarditis or acute pancarditis;
- Untreated adrenal insufficiency or adrenal disorder;
- Terminal illness;
- Participants with rare hereditary problems of galactose intolerance, the Lapp lactase deficiency or glucose-galactose malabsorption;
- Subjects who are participating in ongoing RCTs of therapeutic interventions (including CTIMPs);
- Plan to move out of the region in which the trial is being conducted within the next 2 years.
  1. Statistical Analysis Plan (SAP)
     1. SAP Objectives

The objective of this SAP is to describe the statistical analyses to be carried out for the Final Analysis of the TRUST study.

- - 1. General Principles

Data will be summarised overall and by treatment group. Continuous variables will be summarised as number of observed values, number of missing values, mean and standard deviation, median and interquartile range and minimum and maximum. Categorical data will be summarised as number of observed values, number of missing values, number and percentage in each category.

Recruitment data will be presented by “study centre”, defined as participants recruited in (i) Scotland, (ii) Netherlands, (iii) Ireland, (iv) Switzerland.

Continuous efficacy outcomes involving measurement at follow-up and baseline will be analysed as change from baseline at each time point comparing treatment groups and adjusting for stratification variables and baseline levels of the same variable using linear regression. In addition, data items measured at more than one follow-up time will be analysed using repeated measures regression analyses and in terms of the final assessment for each participant.

Continuous efficacy outcomes measured at final follow-up only will be compared between treatment groups using linear regression adjusting for stratification variables.

For all efficacy outcomes measured at baseline and final visit, additional exploratory analyses that adjust for time from baseline to final visit will be carried out.

Distributions of the residuals will be reviewed and will be taken into consideration in assessing whether or not additional analyses based on transformations should be carried out.

When calculating ThyPRO scores, valid raw total scores containing missing items will be scaled so that the maximum possible score is maintained.

Analyses of haemoglobin levels will be further adjusted for the laboratory at which they were measured.

Time-to-event outcomes will be compared between groups using Cox proportional hazards regression models adjusting for stratification variables. Time to event curves will be based on the Kaplan-Meier method.

Stratification variables are

- Site
- Gender
- Starting dose of levothyroxine

All efficacy analyses will be carried out on the intention to treat population. Safety analyses will be carried out on the ITT population. The main analyses will be modified ITT based on participants with data on the outcome of interest. These analyses will be supported with sensitivity analyses using mixed effects models and multiple imputation. Primary analyses and secondary will be repeated on the per protocol population as exploratory analyses.

The two-sided significance level for each primary outcome is 0.025.

- - 1. Current Protocol

At the time of writing, the current protocol for the TRUST study is version 6.0, dated 19/01/2016.

Future amendments to this protocol will be reviewed for their impact on this SAP, which will be updated only if necessary. If no changes are required to this SAP following future amendments to the study protocol, this will be documented as part of the Robertson Centre Change Impact Assessment processes.

- - 1. Software

Analyses will be carried out using R for Windows v3.0.1, SAS for Windows v9.3, or higher versions of the programs.

1. Analysis
   1. Study Populations

The intention-to-treat (ITT) population consists of all participants randomised excluding those randomised in error.

Modified ITT populations will include ITT participants who have data for the outcome of interest. For analyses at the 12 month visit to be valid, they must have been conducted at 12 months ± 31 days after randomisation.

Per-protocol (PP) populations consists of all participants in the ITT population who

- have data for the outcome variable of interest;
- are on treatment at the time of the analysis being conducted, i.e. they have not withdrawn from treatment and, if they are in the active treatment group, they have not been down-titrated to 0;
- have had their 12 month visit at 12 months ± 31 days after randomisation;
- do not have any other major protocol violation identified prior to database lock.

Reasons for exclusion from PP populations will be summarised.

- 1. Study Status and Consort diagram

A study status table will be created describing the numbers consented and validly randomised, the numbers consented and not validly randomised with reasons.

The table will contain the numbers who are dead, withdrawn from randomised treatment, who have withdrawn consent and are lost to follow-up at the 12 month visit at which the primary outcomes will be assessed, and at the end of the study.

The table will contain the numbers included in the main analysis of the primary outcome for both the ITT and PP populations.

This table will be used to create a CONSORT diagram for the trial.

- 1. Visit Attendance

For each visit, the number and percentage who attended the visit, missed the visit, had completed the study before the visit, had withdrawn from follow-up or had died before the visit will be reported. A visit will be considered as attended if any measurement or participant reported data for this visit are available. A visit will be considered as missed if there are no data for this visit and the participant has not died, not completed the study and not withdrawn from follow-up.

- 1. Baseline Characteristics

The following baseline characteristics will be summarised overall and by treatment group (an additional tabulation will be created for ITT participants by inclusion/exclusion from the modified ITT analysis for the primary outcome):

- age (continuous and categorical as <80 and ≥80), gender, ethnicity, smoking history, alcohol consumption, living arrangements, height;
- cardiovascular risk factors (MI, angina, stroke, TIA, heart failure, PVD, revascularisation, AF, hypertension, diabetes);
- medical history (epilepsy, dementia, osteoporosis, hereditary galactose intolerance, other major illness);
- laboratory measures; TSH, fT4, and haemoglobin at baseline;
- single lead ECG results (heart rate, cardiac rhythm);
- baseline values of outcome measures (see Section 2.5.2.);
- use of concomitant medications, using the WHO ATC classification system level 1 and 2, ordered by ATC code.
  1. Efficacy Outcomes at 12 months
     1. Primary Outcome

Disease-specific quality of life and symptom burden will be analysed as follows in the modified ITT (primary) and PP populations:

- Change from baseline in ThyPRO hypothyroid symptoms score.
  This outcome will be analysed in the modified ITT population as

The estimated treatment effect (p-value and 95%CI) in a linear regression model predicting change from baseline to 12 month visit in ThyPRO hypothyroid symptoms score with the following covariates: randomised treatment, ThyPRO hypothyroid symptoms score at baseline and stratification variables.

- Change from baseline in ThyPRO fatigue score. This outcome will be analysed analogously.
- Statistical significance for each primary outcome requires P ≤ 0.025.
  - 1. Secondary Outcomes

The following secondary outcomes will be analysed at 12 months in the modified ITT (primary) and PP populations

- General QoL (EQ-5D)
- Handgrip strength
- Haemoglobin
- Blood pressure
- Weight, waist circumference and BMI.

These variables will be analysed as for the primary outcomes.

- - 1. Sensitivity analyses

Sensitivity analysis for the modified ITT analyses for the primary and secondary outcomes at 12 months will be carried out using a) multiple imputation of missing values using a model containing age, sex, baseline TSH and any available outcome measurements recorded prior to 12 months and b) using mixed model repeated measures analysis.

- - 1. Subgroup analyses

The primary and secondary outcomes at 12 months will also be analysed in the following subgroups by adding the subgroup variable and its interaction with treatment group:

- Gender (Male/Female)
- Baseline TSH
  - <10 / ≥10
  - <7 / 7-9.99 / ≥10

In addition, continuous interactions with age at randomisation and baseline TSH will be analysed analogously.

- 1. Efficacy outcomes at end of study

The following measurements will be assessed at baseline and the end of study visit:

- ThyPRO hypothyroid symptoms and fatigue scores
- General QoL (EQ-5D)
- Handgrip strength
- Cognitive function (letter digit coding test (LDCT),
- Functional ability (Barthel index, IADL)
- Haemoglobin
- Blood pressure
- Weight, waist circumference and BMI

These outcomes will be analyses as for the primary outcome. An additional sensitivity analysis will be carried out adjusting for the time from baseline to the measurement assessment.

The following outcomes will be assessed at the end of study as described below:

- Comprehensive thyroid quality of life assessment ThyPRO39 at the end of study visit

The estimated treatment effect (p-value and 95%CI) in a linear regression model predicting end of study ThyPRO39 score with the following covariates: randomised treatment and stratification variables.

- Fatal and non-fatal cardiovascular events (confirmed by Endpoint Adjudication Committee)

The estimated treatment effect (p-value and 95%CI) in a Cox proportional hazards models containing randomised treatment group and stratification variables.

- Fatal cardiovascular events (confirmed by Endpoint Adjudication Committee). This outcome will be analysed analogously.
- All-cause mortality. This outcome will be analysed analogously.
  1. Efficacy Outcomes at Other Timepoints

All outcomes assessed post randomisation at timepoints other than baseline, 12 months and end of study will be investigated as change from baseline at these timepoints, analysed as for the primary outcome.

In addition, change from baseline for outcomes analysed at two or more post randomisation visits will be analysed using repeated measures ANOVA models with general covariance structures, first containing only treatment group, study visit, the baseline value of the outcome being assessed and stratification variables and then including a treatment by study visit interaction.

Results at each time point and changes from baseline will also be presented graphically as means and 95% CIs at each time point.

- 1. Safety Outcomes
     1. Study Treatment

Starting dose and dose just before 2, 12, 24, 36 month visits, current dose at final visit and dose at the end of the titration phase in year 1 will be summarised overall and by treatment group.

Time to permanent withdrawal from study treatment (excluding deaths) will presented using Kaplan-Meier time-to-event curves and compared using log-rank statistics. Reasons for permanent withdrawal from study treatment will be tabulated overall and split by treatment group.

- - 1. Withdrawals from the study

Numbers withdrawn from the study and the main reasons for withdrawal will be summarised overall and by treatment group. Kaplan-Meier plots for time to withdrawal will be produced.

- - 1. Serious adverse events

The number and characteristics of SAEs will be summarised. The number and percentage of participants experiencing at least one SAE will be summarised overall and by MedDRA System Organ Class and preferred term. These summaries will be repeated for SAEs at least possibly related to study medication, and for fatal events. Tabulations will be sorted by the MedDRA SOC term order and by preferred term order with in SOCs.

The incidence of other safety outcomes of particular interest (hypothyroidism, atrial fibrillation, heart failure, fractures), will be summarised separately.

Kaplan-Meier plots by treatment for the time to the first occurrence of each safety outcome of particular interest will be produced. Time to event will be compared between groups using log-rank tests. If there are sufficient events, Cox proportional hazards regression adjusting for stratification variables will be used.

- - 1. Concomitant medications

The number and percentage of participants receiving at least one concomitant medication will be summarised by ATC codes level 1 and 2.

- - 1. Other safety data
- ThyPRO hyperthyroid symptoms will be summarised at each time point, overall and by treatment group. Change in ThyPRO hyperthyroid symptoms will be compared between treatment groups in the same way as the primary analysis.
- A listing of deaths will be provided.

1. Document History

This is version 1.0 (initial creation) of the SAP for the final analysis for TRUST, dated 20/05/2016.

1. Tables and Figures

The layout of the tables and figures will be finalised based on tables created using dummy treatment codes prior to database lock.

1. Listings

- All SAEs will be listed.
